# Seroprevalence and risk factors associated with bluetongue and Schmallenberg virus infections in domestic small ruminants in Türkiye

**DOI:** 10.1007/s11250-026-04951-9

**Published:** 2026-03-16

**Authors:** Feride Firdevs Ertarğın, Ali Riza Babaoglu, İsmail Hakkı Ekin

**Affiliations:** 1 Department of Virology, Graduate School of Health Sciences, Van Yuzuncu Yil University, Van, Türkiye; 2 Department of Virology, Faculty of Veterinary Medicine, Aksaray University, Aksaray, Türkiye; 3 Department of Microbiology, Faculty of Veterinary Medicine, Van Yuzuncu Yil University, Van, Türkiye

**Keywords:** Bluetongue virus, c-ELISA, Risk factors, Schmallenberg virus, Seroprevalence, Sheep, Goat, Türkiye

## Abstract

Bluetongue virus (BTV) and Schmallenberg virus (SBV) are Culicoides-borne viruses that cause major reproductive and economic losses in ruminants, with their spread shaped by climate-driven vector activity and growing livestock movements. This cross-sectional study assessed the seroprevalence and risk factors for both viruses in small ruminants in eastern Türkiye. A total of 596 sheep and goats from 65 farms across 11 districts were sampled between October 2024 and June 2025 and tested using commercial competitive enzyme-linked immunosorbent assays (c-ELISA). Statistical analyses were performed using SPSS, and chi-square tests were applied to assess associations between BTV and SBV seroprevalence and various potential risk factors. Overall, 69.8% (95% CI: 65.94%–73.46%) of the animals were seropositive for BTV, with similar rates in sheep and goats. District-level variation showed a trend toward statistical significance (*P* = 0.053), indicating possible spatial heterogeneity in BTV exposure. Contact with cattle was the only significant predictor of BTV seropositivity (aOR = 2.35), highlights the importance of cattle in the local transmission cycle. No associations were observed between species or sex. SBV antibodies were detected in only two animals (0.33%), precluding meaningful statistical analysis. These findings confirm that BTV remains endemic in eastern Türkiye and highlight the need for integrated multispecies surveillance, whereas the very low SBV seroprevalence underscores the importance of periodic monitoring under shifting environmental conditions.

**Supplementary Information:**

The online version contains supplementary material available at 10.1007/s11250-026-04951-9.

## Introduction

Bluetongue virus (BTV) and Schmallenberg virus (SBV) are non-contagious, vector-borne RNA viruses that affect a wide range of domestic and wild ruminants, including sheep, goats, cattle, camels, and deer. Both viruses are primarily transmitted by *Culicoides* biting midges (Diptera: *Ceratopogonidae*) and share similar epidemiological patterns, particularly pronounced seasonality driven by climatic conditions that favor vector activity (Beer and Wernike 2021; Al-Tubi et al. 2024). BTV belongs to the genus *Orbivirus* within the family *Sedoreoviridae*, and SBV is classified under the genus *Orthobunyavirus* of the family *Peribunyaviridae* and the *Schmallenberg orthobunyavirus* species (Hoffmann et al. 2012; ICTV 2017; ICTV 2023). Despite their genetic differences, both viruses share broad host ranges—most notably in cattle, sheep, and goats—and are capable of establishing viremia that enables efficient transmission to insect vectors. Clinically, they are associated with reproductive and developmental disorders, including reduced fertility, stillbirth, and congenital malformations (Thabet and Lajnef 2024).

Ecologically, they exhibit comparable behaviors with the capacity to spread into new regions under shifting environmental and climatic conditions. Modeling studies indicate that vector dynamics play a key role in BTV transmission, highlighting its relevance as an important arboviral threat to animal health and productivity (Tabachnick 2010; Beer et al. 2013; Maclachlan et al. 2015; Đurić et al. 2018). Within the One Health framework, arboviral diseases arise from complex interactions among animal hosts, vectors, humans, and environmental factors, underscoring the need for integrated surveillance systems. Coordinated monitoring across sectors is essential for early detection and effective control of vector-borne infections, particularly under changing climatic conditions. As emphasized by Massengo (2023), cross-sectoral surveillance at the human–animal–environment interface is critical for managing complex infection dynamics, highlighting the importance of systematic monitoring of livestock arboviruses such as BTV and SBV.

BTV is endemic in parts of Africa, the Middle East, and Asia. The World Organization for Animal Health (WOAH) recognizes 24 notifiable serotypes, and 36 classical and atypical serotypes have been identified to date (WOAH 2021; Thabet and Lajnef 2024). Importantly, some atypical strains, including BTV-25, BTV-26, and BTV-27, exhibit distinct epidemiological characteristics, such as limited or absent vector dependence and the ability to establish persistent infections, particularly in goats, which may facilitate silent circulation and complicate surveillance and control efforts (Beyan et al. 2025). Although many domestic and wild ruminants are susceptible, disease severity varies by host and strain; sheep are generally more affected, and maternal infection can lead to abortion or fetal defects (Zeiske et al. 2025). The clinical signs range from acute to subclinical, with the acute form marked by fever, facial swelling, oral ulcers, and cyanosis. This infection also has major economic impacts, including mortality, productivity losses, control and vaccination costs, and trade restrictions (Saminathan et al. 2020).

Diagnosis relies on both molecular and serological approaches. RT-PCR and virus isolation are the primary methods used to detect viral RNA in samples. Common serological assays include agar gel immunodiffusion (AGID), various enzyme-linked immunosorbent assays (ELISAs), immunofluorescence (IF), and virus neutralization (VN). Among these, competitive ELISA (c-ELISA) offers notably high specificity (99.6%) and greater sensitivity compared with other ELISA methods. The World Organisation for Animal Health (WOAH) Manual of Diagnostic Tests and Vaccines recommends AGID and c-ELISA, emphasizing the latter as a rapid and reliable test capable of detecting antibodies as early as six days post-infection (Breard et al. 2004; Rojas et al. 2019; WOAH 2021).

BTV is a notifiable disease in Türkiye, as in several European countries. The first recognized outbreak occurred in Hatay between 1944 and 1947 and was subsequently brought under control through strict intervention measures. Nevertheless, subsequent serological and virological studies have identified multiple BTV serotypes in different ruminant species across the country, indicating ongoing endemic circulation of the virus (Yılmaz and Ozkul 2012; Yılmaz et al. 2015; Çelik and Şahin 2019; Bayram and Gümüşova 2021). In eastern Türkiye, available evidence suggests ongoing BTV circulation driven by extensive small ruminant husbandry and frequent animal movements; however, systematic, province-level seroepidemiological data remain largely unavailable. Collectively, these findings highlight the continued epidemiological relevance of BTV and the need for sustained and regionally targeted surveillance, particularly in areas with high animal densities.

SBV was first identified in Germany in 2011 and rapidly spread across Northern Europe, reaching the British Isles, Eastern Europe, and the Mediterranean, where it became endemic within a short period of time (Hoffmann et al. 2012; Wang et al. 2025). Belonging to the Simbu serogroup of the genus *Orthobunyavirus*, SBV is genetically close to Akabane virus and appears to enhance its adaptability through frequent genomic recombination (Hughes et al. 2020). Although Europe is the primary endemic region, recent seroepidemiological studies demonstrate an upward trend in SBV activity, and its detection in sheep shows that the geographical spread of the virus now extends beyond Europe. SBV has been detected serologically in Africa, the Middle East, East Asia, and Malaysia, with seroprevalence approaching 50% in some areas, indicating broad global circulation (Zhai et al. 2018; Wang et 2025). Its transmission relies mainly on *Culicoides* midges, in which the virus replicates during the extrinsic incubation period before it becomes transmissible. The detection of viral RNA in nulliparous midges suggests possible transovarial transmission, potentially supporting overwintering and seasonal re-emergence (Larska et al. 2013).

SBV can be vertically transmitted in ruminants, infecting the fetus during maternal viremia and leading to abortion, stillbirths, or congenital malformations. Experimental infection studies in pregnant goats have demonstrated that fetal susceptibility in small ruminants occurs during early gestation, particularly around days 28 to 42 of pregnancy, when transplacental infection can result in fetal death and central nervous system lesions; detection of SBV RNA in malformed fetuses provides strong evidence for this vertical transmission route (Laloy et al. 2017). Horizontal transmission via oral or nasal secretions is unlikely to occur under natural conditions. Adult cattle usually show mild, transient signs, and most sheep and goats remain subclinical, while infection during pregnancy may lead to severe fetal deformities, such as arthrogryposis and hydrocephalus (Wernike et al. 2013; De Regge et al. 2013).

The diagnosis of SBV relies on molecular tests for acute infection and serology for monitoring. RT-PCR and virus isolation are the primary methods for detecting viral RNA in the blood, fetal tissues, and/or the central nervous system. Serological approaches include indirect and competitive ELISAs, indirect immunofluorescence, and virus neutralization tests (VNT). Although VNT is the reference method, it is labor-intensive, whereas ELISAs enable high-throughput screening (WOAH 2012). A commercial indirect ELISA shows strong agreement with VNT (approximately 98–99%), supporting its suitability for large-scale surveillance (Bréard et al. 2013).

In Türkiye, SBV gained attention following the 2011 outbreaks in Europe, and its circulation within the country was confirmed in 2014 through the detection of the viral genome in aborted or low-viability ruminant fetuses (Yilmaz et al. 2014). Subsequent serological investigations have reported high seropositivity rates in both domestic and wild ruminants, demonstrating that the virus continues to circulate silently across various regions (Elmas et al. 2018; Yılmaz et al. 2024). However, in eastern Türkiye, available data on SBV remain limited, and no comprehensive, province-level seroepidemiological assessments have been conducted to date. Discussion of vaccination programs is especially important in areas with intensive livestock farming, as is the need for ongoing epidemiological surveillance of the disease.

Together, the continuous circulation of BTV and SBV in Türkiye, their shared vector-borne nature, and their capacity to cause substantial reproductive and economic losses highlight the need for updated epidemiological data in small ruminants. Van Province represents a particularly suitable setting for such investigations, not only because of its large small-ruminant population but also due to its strategic border location with Iran, which facilitates transboundary animal movements. In addition, the province is characterized by a continental climate, marked seasonal variation, wetlands and irrigated areas around Lake Van, and widespread transhumant and intensive production systems, all of which favor the establishment and seasonal activity of Culicoides vectors. Despite evidence of viral activity, comprehensive field-based assessments remain limited prior to the introduction of vaccination programs. Therefore, the present study aims to determine the seroprevalence of BTV and SBV in domestic small ruminants in Van Province in eastern Türkiye and to identify key associated risk factors. These findings are expected to support evidence-based surveillance strategies and improved control measures for both arboviruses in regions with intensive livestock production systems.

## Materials and methods

### Study area and ethical statement

**Fig. 1:**
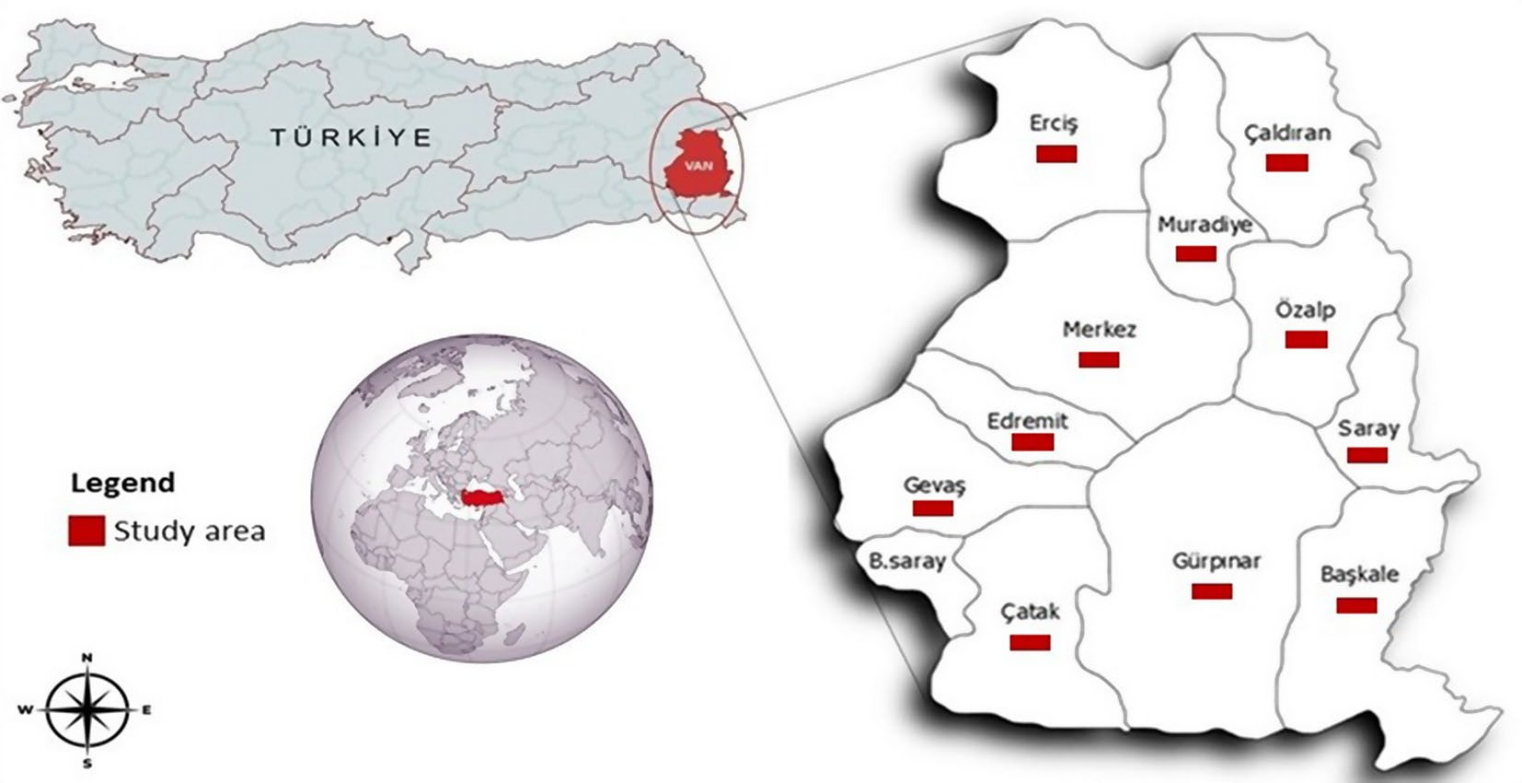
Map showing the geographical position of the study province and the locations of sampled districts

Van Province, situated on the Eastern Anatolia Plateau bordering Iran, features a continental climate with long cold winters and short dry summers. Its mountainous terrain, volcanic plateaus, and the basin of Lake Van, the world’s largest soda lake, create ideal conditions for small ruminant farming. This plateau, encompassing the provinces of Van, Muş, Ağrı, Erzurum, and Bitlis, is characterized by extensive meadows and grasslands and by small-scale small-ruminant farming for meat and milk production. According to the most recent livestock statistics, the small ruminant population (sheep and goats) in Türkiye reached 58.2 million as of June 2025, approximately 83% of which are female. In the Eastern Anatolia region, where small ruminant production is particularly intensive, Van Province alone accounts for more than 3.3 million animals (Turkish Ministry of Agriculture and Forestry, 2025). This study was conducted in 11 districts (Başkale, Çalıdıran, Çatak, Edremit, Erciş, Gevaş, Gürpınar, Merkez, Muradiye, Özalp, and Saray) out of 12 districts representing the province’s various climatic and geographical conditions (Fig. 1). Due to adverse climatic conditions, sampling could not be conducted in one district (Bahçesaray) during the study period. Sheep production in the region is dominated by fat-tailed local breeds, particularly Morkaraman (Akkaraman type), which are well adapted to extensive grazing systems and the harsh climatic conditions of Eastern Türkiye. All procedures involving animals were conducted in accordance with the ethical guidelines of the Van Yuzuncu Yil University Local Ethics Committee for Animal Experiments (Decision no: 2025/01–02).

### Sampling method

To determine the sample size required for assessing the seroprevalence of BTV and SBV in small ruminants, the formula described by Thrusfield (2018) was applied as follows: N = Z^2^ P_exp_ (1-P_exp_)/d^2^

Where: 

**N** = required sample size,

**P**_**exp**_ = expected prevalence (assumed at 50%, 0.5).

**Z** = standard normal deviate for a 95% confidence interval (1.96)

**d** = desired margin of error (5% or 0.05).

In the absence of recent and reliable prevalence data for BTV and SBV in small ruminants, an expected prevalence of 50% was used as a conservative assumption to maximize sample size and ensure adequate statistical power. Based on this assumption, the minimum required sample size was calculated as 384 animals using Thrusfield’s formula; given the large regional population (~ 3.3 million animals), application of a finite population correction did not alter this estimate. To further improve the precision and reliability of seroprevalence estimates, accommodate heterogeneity across districts, and strengthen the robustness of risk factor analyses, the final sample size was increased to 596 animals.

Samples were collected by experienced veterinarians using standard procedures to minimize animal stress, and animal owners were informed about the study prior to sampling. Data were obtained through a standardized questionnaire administered to owners to capture factors potentially associated with BTV or SBV infection. The questionnaire was not formally pre-tested or validated and was intentionally kept concise to ensure feasibility and farmer compliance, focusing on core variables only (location, species, sex, and contact with cattle).

Blood samples were collected between October 2024 and June 2025 from 596 apparently healthy small ruminants (563 sheep and 33 goats) aged over one year, raised under intensive and family based transhumant systems on 65 farms across 11 districts of Van province (Başkale, Çalıdıran, Çatak, Edremit, Gevaş, Gürpınar, Merkez [İpekyolu], Muradiye, Özalp, and Saray). Of the 596 animals, 558 were females (93.6%) and 38 were males (6.4%). Species-specific sex distributions were as follows: sheep, 526 females and 37 males; goats, 32 females and 1 male. Based on farm records and regional livestock structure, the sampled sheep predominantly consisted of local fat-tailed breeds typical for Van Province, mainly Morkaraman (Akkaraman type).

Due to the lack of reliable individual records and field constraints across multiple districts, detailed age stratification was not feasible. Animals younger than one year were excluded to minimize the potential influence of maternally derived antibodies and to ensure that detected seropositivity reflected natural exposure. Farms were selected based on accessibility and owner consent, and animals were randomly selected within each participating farm, with approximately 10–25% of animals sampled per farm; each animal was sampled once to reflect cumulative serological exposure in this cross-sectional design. The distribution of sampled animals reflects regional livestock demographics, with sheep populations approximately five times larger than goats, as well as field constraints related to farm access and owner consent. Sampling therefore depended on animal availability and owner permission.

As there is currently no licensed SBV vaccine in Türkiye and no national vaccination program for BTV, none of the animals in our sample had a vaccination history against either virus. The proportion of animals sampled from each farm ranged from 10% to 25%. Blood was drawn from the jugular vein into vacuum tubes for serum collection. The blood samples were centrifuged at 2,000 rpm for 10 min, and the sera transferred to stock tubes were heated at 56 °C for 30 min to inactivate endogenous complement and kept at -20 °C until analysis (Table 1).

**Table 1:** Seroprevalence of BTV in small ruminants in relation to different risk factors (with 95% confidence intervals)

Variable	Category	Animals Tested (*n*)	BTV_Ab positive (*n*)	BTV Seroprevalence (%)	BTV 95% CI (%)
District	Başkale	42	31	73.8	58.0–86.1
	Çaldıran	22	15	68.2	45.1–86.1
	Çatak	11*	9	81.8	48.2–97.7
	Edremit	7*	3	42.9	9.9–81.6
	Erciş	35	28	80.0	63.1–91.6
	Gevaş	15*	10	66.7	38.4–88.2
	Gürpınar	39	34	87.2	72.6–95.7
	Merkez(İpekyolu)	75	51	68.0	56.2–78.3
	Muradiye	20	17	85.0	62.1–96.8
	Özalp	312	203	65.1	59.5–70.3
	Saray	18*	15	83.3	58.6–96.4
Species	Sheep	563	393	69.8	65.8–73.6
	Goat	33	23	69.7	51.3–84.4
Sex	Male	38	26	68.42	51.3–82.5
	Female	558	390	69.90	65.9–73.7
Contact Cattle	No	323	199	61.6	56.3–67.1
	Yes	273	216	79.12	73.8–83.8
Total		596	416	69.8	65.9–73.5

* District-level seroprevalence estimates based on small sample sizes (*n* < 20) should be interpreted with caution due to wide confidence intervals

### Competitive enzyme‑linked immunosorbent assay (c‑ELISA)

A competitive enzyme-linked immunosorbent assay (c-ELISA) was employed to differentiate BTV and SBV from other closely related viruses, including discrimination of BTV from Epizootic Hemorrhagic Disease Virus (EHDV) and *Schmallenberg orthobunyavirus* species from other closely related members within the *Peribunyaviridae* family. All serum samples were screened using a commercial c-ELISA kit (ID Screen^®^ bluetongue competition ELISA IDvet, Grables, France; catalog/lot: BTC-5P/303 − 028) for the presence of antibodies specific to the BTV VP7 protein (Breard et al. 2004) and a commercial c-ELISA kit (ID Screen^®^ Schmallenberg virus Competition Multi-species ELISA IDvet, Grables, France; catalog/lot: SBV-5P/Q08) for the detection of antibodies directed against the SBV nucleoprotein (Bréard et al. 2013). These assays have been reported to exhibit high diagnostic sensitivity (100%) and specificity (99%) previously (Breard et al. 2004; Rojas et al. 2019; Bréard et al. 2013). Competition percentages were calculated as the signal-to-noise ratio (S/N%) using the following formula:

S/N% = (OD_sample_ / OD_NC_) × 100. Interpretation of the results followed the manufacturer’s cutoffs. For BTV, samples with S/N% < 40% were considered positive and S/N% ≥ 40% were considered negative. For SBV, S/N% < 40% was interpreted as positive, 40% ≤ S/N% < 50% as doubtful, and S/N% ≥ 50% as negative.

### Potential risk factors and statistical analysis

Data were collected using a structured form that included information on location, species (sheep or goat), sex, and contact with cattle (yes or no).

Statistical analyses were conducted using SPSS software (IBM SPSS 24, USA). Because animals were sampled within farms, observations were not fully independent; farm-level clustering was not explicitly adjusted for and is therefore acknowledged as a limitation of the study. The chi-square (χ²) test was applied to evaluate the association between the seroprevalence of BTV and SBV infections and various potential risk factors. Following this, a logistic regression analysis was performed to examine the relationship between the presence of BTV and SBV antibodies and risk factors such as species, sex, contact with cattle (yes or no), and district. Variables with a P value < 0.25 in univariate analysis were subsequently considered for inclusion in multivariable logistic regression models, in accordance with common epidemiological practice to avoid premature exclusion of potentially important predictors at the screening stage. Prior to multivariable analysis, multicollinearity among candidate variables was evaluated by examining correlation matrices and variance inflation factors (VIFs), and no evidence of problematic multicollinearity was detected. This model was then performed to assess the association between seropositivity and selected risk factors, with odds ratios (ORs) and 95% confidence intervals (CIs) estimated for variables retained in the final model.

## Results

All serum samples (563 from sheep and 33 from goats) were tested for antibodies against BTV and SBV using commercial competitive ELISA (c-ELISA) kits; the number of animals sampled per district varied widely, ranging from 7 to 312, reflecting differences in herd size and farm availability across the study area. Of these, 416 samples (69.8%) were positive for BTV-specific antibodies, with a 95% confidence interval (CI) of 65.94%–73.46%. Only 2 samples (0.33%) tested positive for SBV-specific antibodies, with a 95% CI of 0.04%–1.21%.

BTV antibodies were detected in both sheep and goats, with a comparable prevalence of 69.8% (393/563) in sheep and 69.7% (23/33) in goats. There was no statistically significant difference in BTV seroprevalence between species (χ² = 0.00, *P* = 1.000) and between males and females (χ² = 0.00007, *P* = 0.993). The markedly lower number of goats reflects the underlying livestock population structure in the study area and field-based sampling constraints. Overall sample distribution and species-specific results are summarized in Table 1.

SBV antibody detection was limited to two sheep (0.36%, 2/563 at the sex level) and was absent in goats (0%). Due to the very low number of SBV-seropositive animals (*n* = 2), risk factor analysis for SBV was not performed. In addition, none of the tested samples were classified within the doubtful range (40% ≤ S/N% < 50%).

BTV-specific antibodies were detected in all districts studied, with seroprevalence ranging between 42.9% in Edremit to 87.2% in Gürpınar. The highest rates were observed in Gürpınar (87.2%), Muradiye (85.0%), and Saray (83.3%). District-level differences were evaluated using an overall chi-square test to assess geographic variation in BTV exposure, and no pairwise post hoc or multiple comparisons between individual districts were performed. The association between district and BTV seropositivity was borderline significant (χ² = 18.09; df = 9; *P* = 0.053), indicating possible geographic variation in exposure among the sampled areas. A summary of BTV serological outcomes and district-level distributions is presented in Table 2.

**Table 2:** Univariate chi-square analysis of factors associated with BTV seropositivity

Variable	χ²	*p* (approx.)	Interpretation
Contact with cattle	20.19	0.000022*	Significant
District	18.09	0.0535*	Borderline significance (≈ 0.05)
Species	0.0000	1.000	Not significant
Sex	0.00007	0.993	Not significant

* The result was significant if P value was less than 0.05

Additionally, BTV seroprevalence was higher in animals reported to have had contact with cattle; it increased from 58.3% to 100% in goats and from 62.1% to 78.4% in sheep (Table 3). SBV antibodies were detected in two animals from Başkale (2/42, 4.8% at the district level). All other districts were SBV-negative. Due to this extremely low number of SBV-positive samples, risk factor analysis for SBV was not statistically meaningful and was therefore not performed.

**Table 3:** Comparative seroprevalence of BTV by species and contact with cattle

Species	Contact with cattle	Total	BTV_pos	BTV_seroprevalence %
Goat	No	24	14	58.33
Goat	Yes	9	9	100.0
Sheep	No	298	185	62.08
Sheep	Yes	264	207	78.40

Univariate chi-square analysis of potential risk factors for BTV seropositivity (χ² = 20.19; *P* < 0.001) identified contact with cattle as the only statistically significant variable (*P* < 0.001), while species and sex were not significantly associated with BTV serostatus. District showed borderline significance (*P* = 0.053). Results of the univariate risk factor analysis are summarized in Table 2.

**Fig. 2:**
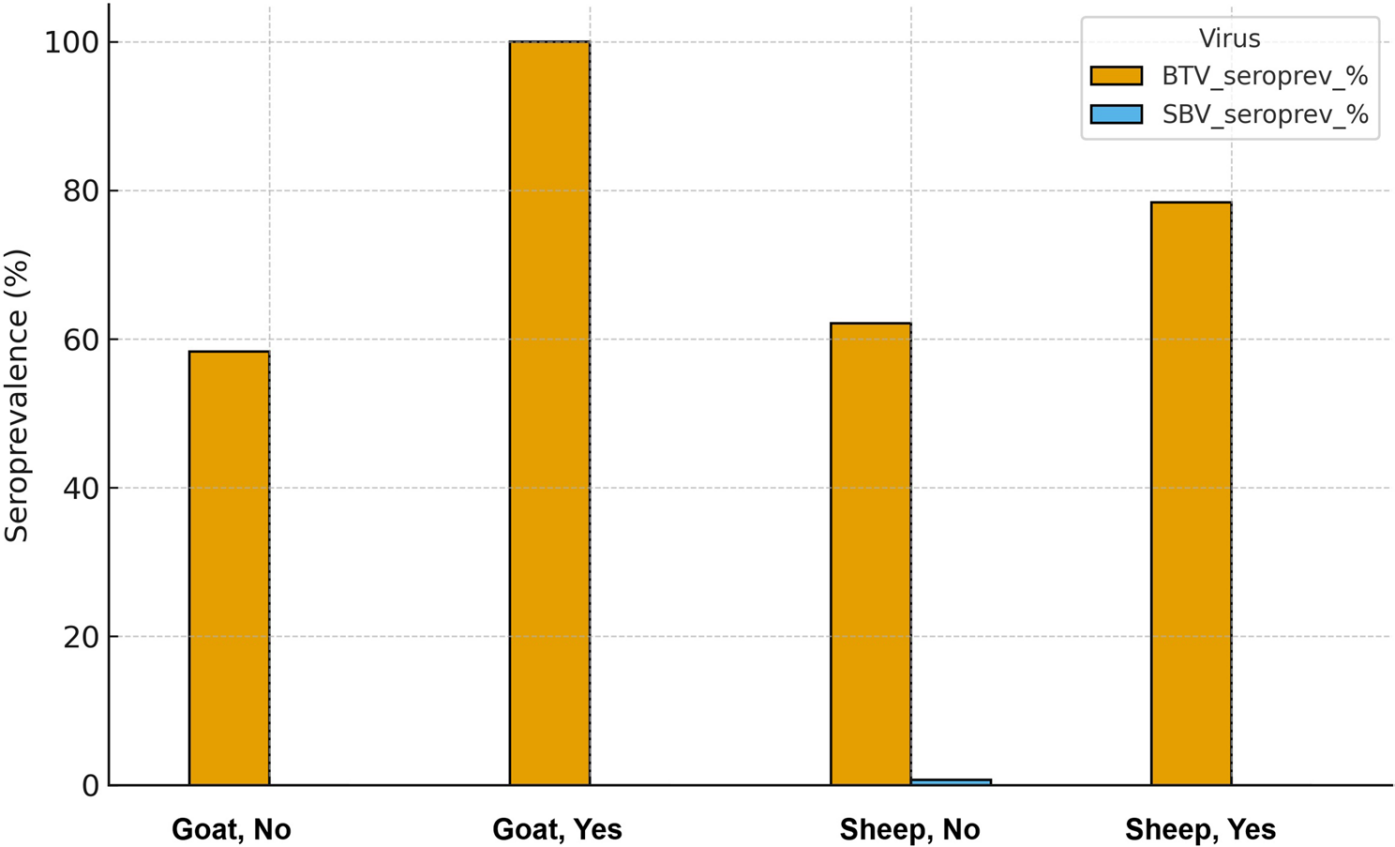
Seroprevalence of BTV and SBV in small ruminants by species and contact with cattle

Variables with *P* < 0.25 in univariate analysis (contact with cattle and district) were entered into the multivariable logistic regression model; after adjustment, only contact with cattle remained a statistically significant predictor of BTV seropositivity, while district-level odds ratios were elevated but not significant (Table 4) (Fig. 2).

**Table 4:** Multivariable logistic regression analysis of risk factors associated with BTV seropositivity

Variable	Coefficient (β)	OR	95% CI (Lower–Upper)	*p*-value
Intercept	—	—	—	—
Contact with cattle*	0.855	2.35	1.60–3.45	< 0.001
District (overall)*	—	—	—	0.053

β = logistic regression coefficient; OR = odds ratio; CI = confidence interval

* District was included as a categorical variable, and the reported P value represents the overall (global) effect of district. Reference categories were “no contact with cattle” and the district with the lowest observed BTV seroprevalence

## Discussion

Global climate change and increased animal movement are key drivers shaping the epidemiology of arboviruses such as BTV, SBV, and Akabane virus by extending vector activity and transmission efficiency (Hart et al. 2025; Fairbanks et al. 2024). Climate variability and livestock trade have been widely recognized as major contributors to orbivirus dissemination worldwide (Barua et al. 2025). In Türkiye, favorable ecological conditions combined with intensive livestock production systems support sustained arbovirus circulation. Accordingly, the detection of antibodies against BTV and SBV in the present study is consistent with recent national reports and highlights the need for targeted surveillance and strengthened vector control, particularly for BTV in high-risk regions (Bilge-Dağalp et al. 2018; Doğan et al. 2022; Yılmaz et al. 2024).

This study demonstrated a high overall seroprevalence of BTV in small ruminants, whereas SBV seroprevalence was negligible. BTV exposure was similar in sheep and goats and showed no association with sex, indicating broadly similar infection patterns across host categories. Given that sheep and goats in the study area are typically managed together under similar grazing and housing systems, both species are likely exposed to comparable levels of *Culicoides* vectors. However, the apparent similarity in seroprevalence should be interpreted with caution, as the very small goat sample size limits statistical precision and may contribute to sampling-related similarity. District-level prevalence varied from 42.9% to 87.2%. Contact with cattle was the only risk factor for BTV seropositivity, and remained significant in the multivariable model. In line with the study objectives, these findings address the evidence gap regarding contemporary BTV and SBV exposure and associated farm-level risk factors in eastern Türkiye. Consistent with the Results, district was not retained as a statistically significant independent predictor after adjustment and is therefore interpreted cautiously. Due to the small number of SBV-positive animals (*n* = 2), risk-factor analysis for SBV was not feasible.

The high BTV seroprevalence (~ 70%) observed in this study is consistent with earlier reports from Türkiye, which indicate substantial regional variability ranging from 0% to 87.5%. Findings from Samsun (0%) (Albayrak et al. 2012), Siirt (73.1%) (Çelik and Şahin 2019), the Marmara region (38.7%) (Pestil and Bulut 2024), and Kars (10.65%) (Yılmaz et al. 2015) illustrate the heterogeneous nature of BTV circulation and collectively confirm the widespread presence of BTV among small ruminant populations in the study area. Similar exposure patterns between sheep and goats in our study may reflect shared vector pressures, livestock practices, or sampling characteristics. In contrast, SBV seroprevalence was extremely low (0.33%), consistent with previous reports where small ruminants show much lower SBV exposure compared with cattle (Elmas et al. 2018; Dogan et al. 2022; Yılmaz et al. 2024; Dagnaw et al. 2024). Collectively, these findings indicate that SBV circulation in Türkiye’s small ruminant population is sporadic or minimal relative to BTV.

The association between contact with cattle and BTV seropositivity underscores the role of cattle as key amplifying hosts. Their prolonged viraemia, combined with higher attractiveness to Culicoides vectors, increases infection pressure on nearby sheep and goats. These ecological dynamics emphasize the need for integrated, multispecies management strategies incorporating cattle into surveillance, vector control, and farm planning rather than focusing on small ruminants alone (Bonneau et al. 2002; Saminathan et al. 2020). Consistent with some previous studies, we found no significant differences in BTV seropositivity by species or sex, suggesting broadly uniform exposure in this setting (Abera et al. 2018; Abebe et al. 2023). The presence of seropositive animals with and without cattle contact indicates that BTV transmission is not exclusively cattle-dependent and can be maintained through vector-mediated transmission among small ruminants. Because sampling spanned multiple seasons (October 2024 to June 2025), the observed seroprevalence likely reflects cumulative exposure, and seasonal variation in Culicoides activity and sampling timing across districts may have contributed to some of the observed district-level differences. Although BTV seropositivity showed numerical variation across districts, this association did not reach statistical significance (*P* = 0.053) and is therefore interpreted cautiously as a hypothesis-generating observation rather than evidence of true geographical clustering, consistent with previous studies highlighting that apparent spatial heterogeneity may arise from sampling structure and analytical limitations (Ma et al. 2019; Charron et al. 2013).

The very low SBV seroprevalence (0.3%) observed in this study suggests limited virus activity in the study area and may reflect factors such as sampling period, local vector ecology, host species composition, or low-level viral circulation. Similarly low SBV seropositivity has been reported in small ruminants in Türkiye (Elmas et al. 2018; Yılmaz et al. 2024; Azkur et al. 2013), and exposure is generally lower in sheep and goats than in cattle (Dagnaw et al. 2024), consistent with the absence of meaningful SBV risk factor associations. In contrast, Akabane virus (AKAV) seroprevalence has been reported to range from 0% to 42% in different regions of Türkiye (Doğan 2018; Özsoy et al. 2021), indicating more established circulation of AKAV, whereas the low SBV seropositivity detected here is more consistent with sporadic or limited local transmission, underscoring the need for continued surveillance.

Beyond ecological and management-related factors, arboviral infections also elicit distinct host immune responses that may influence susceptibility and disease outcomes. Various host-related biomarkers, including inflammatory cytokines and immunological indicators, have been proposed to improve understanding of disease pathogenesis, prognosis, and surveillance strategies for arboviruses such as BTV, dengue, Zika, West Nile, and chikungunya viruses (Adekola et al. 2024). Although such host inflammatory and hematological parameters were not assessed in the present study, their integration into future investigations could provide valuable insights into host–pathogen interactions and complement seroepidemiological findings for arboviral infections.

A key strength of this study is its large and geographically diverse sample (*n* = 596), allowing reliable estimation of BTV and SBV seroprevalence at the regional level under real-world field conditions. In addition, the use of c-ELISA kits with high diagnostic performance (99–100% sensitivity and specificity) ensured consistent antibody detection and minimized misclassification. Such large-scale, field-based datasets align with advanced epidemiological approaches, as multicenter and real-world data frameworks have been shown to enhance the robustness, external validity, and translational relevance of infectious disease surveillance studies (Zhuang et al. 2025).

However, several limitations should be considered. The cross-sectional design and single-time-point sampling prevent causal inference and assessment of temporal or seasonal variation in BTV transmission. A further limitation is the marked imbalance in sample size between sheep and goats, reflecting regional livestock demographics and field constraints. Accordingly, goat-related findings should be interpreted with caution, as the results primarily reflect the epidemiological situation in sheep, and future targeted sampling will be needed to obtain more representative data for goats. Risk factor analysis relied on a limited questionnaire including only four variables, and no adjustment was made for farm-level clustering, which may have led to underestimated standard errors and inflated borderline district associations. In addition, some districts were undersampled, reducing statistical power and limiting spatial inference, while non-random farm selection may have introduced selection bias. Contact with cattle was assessed solely by farmer report without independent verification or quantitative characterization, potentially leading to exposure misclassification. The very low number of SBV-positive samples precluded meaningful risk factor analysis. Future longitudinal, cluster-adjusted studies with expanded risk factor data, balanced spatial sampling, inclusion of vector- and management-level variables, and molecular characterization are therefore warranted.

Overall, our findings confirm that BTV remains highly endemic among small ruminants in eastern Türkiye. In Van Province, this highlights the need for integrated, multi-species control strategies that prioritize targeted vaccination in districts with higher seroprevalence, together with enhanced Culicoides vector surveillance in ecologically high-risk areas. In addition, the implementation of biosecurity measures tailored to herd management systems, including regulation of shared grazing areas and housing practices that reduce vector exposure, would further support effective and sustainable BTV control in this endemic setting. Although SBV circulation was minimal, its recognized reproductive impacts and sensitivity to environmental shifts, such as climate warming, irrigation, and dam construction justify periodic monitoring and its inclusion in diagnostic investigations of reproductive disorders. By demonstrating widespread BTV exposure alongside very limited SBV activity, this study underscores the importance of adaptive, risk-based surveillance frameworks. Beyond animal health, such surveillance data provide critical inputs for evidence-based risk assessment and integrated control strategies, supporting preparedness and decision-making at the animal–human interface (Zhuang et al. 2023).

## Conclusion

This study demonstrates widespread exposure to BTV among small ruminants in Van Province, whereas SBV activity appears to be limited. These findings underscore the continued epidemiological importance of BTV in the region and support the implementation of targeted, risk-based surveillance, vaccination, and control strategies for vector-borne viral diseases in small ruminants. Key limitations include the cross-sectional design, the restricted scope of risk factor data, and the lack of adjustment for farm-level clustering. Consistent with the aims of this study, these results provide updated regional evidence to inform risk-based surveillance and control strategies. Future research should adopt longitudinal and cluster-adjusted designs and incorporate expanded epidemiological variables, including herd characteristics, vector dynamics, and farm-level biosecurity practices, to better elucidate transmission patterns and inform more effective disease prevention efforts.

## Supplementary Information

Below is the link to the electronic supplementary material.

Supplementary Material 1

Supplementary Material 2

## Data Availability

All data generated or analyzed during this study are presented within the article, and additional data can be obtained from the corresponding author upon reasonable request.
