# Supplementary material for: Seroprevalence and risk factors associated with bluetongue and Schmallenberg virus infections in domestic small ruminants in Türkiye
Source: Trop Anim Health Prod. 2026 Mar 16;58(3):181. doi: 10.1007/s11250-026-04951-9 (PMC12992406; doi:10.1007/s11250-026-04951-9)
Supplement: Supplementary file 1 — Supplementary Material 1 [file 11250_2026_4951_MOESM1_ESM.pdf]

Evrak Tarih ve Sayısı: 06.02.2025-652804

T.C.
VAN YÜZÜNCÜ YIL ÜNİVERSİTESİ REKTÖRLÜĞÜ
Hayvan Deneyleri Yerel Etik Kurulu

Sayı : E-27552122-604.01-652804 06.02.2025
Konu : Dr. Öğr. Üyesi Ali Rıza BABAOĞLU'na ait Araştırma Onay Gerektirmeyen Belge

Sayın Dr. Öğr. Üyesi Ali Rıza BABAOĞLU

Hayvan Deneyleri Yerel Etik Kurulu'nun 31/01/2025 tarih ve 2025/01-02 sayılı kararı gereği; Yürütücülüğünü yapmayı tasarladığınız, "Van İlindeki Küçük Ruminatlarda Abortusa Neden Olan Bazı Viral Etkenlerin Seroprevalansının Araştırılması" adlı çalışmayla ilgili, Van YUHADYEK Çalışma Usül ve Esaslarına Dair Yönergenin 6. Maddesinin 8. Fıkrasının m-2 bendinde yer alan "Ölü hayvan veya dokusu, mezbaha materyalleri, atık fetuslar ile yapılan prosedürler" hükmü gereğince VAN YUHADYEK'ten Çalışma ve Araştırma Kesin Sonuç Onay Belgeleri alınmasına gerek olmadığına karar verilmiştir.

Bilgilerinize rica ederim.

Prof. Dr. Semiha DEDE
Etik Kurulu Başkanı

Ek: 02 (1 Sayfa)

Bu belge, güvenli elektronik İmza ile imzalanmıştır.
Doğrulama Kodu :BSCEN7BF53 Pin Kodu :86042 Belge Takip Adresi : https://www.turkiye.gov.tr/vyy-ebys

Adres:Van Yüzüncü Yıl Üniversitesi Hayvan Deneyleri Yerel Etik Kurulu Zeve Kampüsü 65080 Tuşba / Van
Telefon No:+90 432 2251701-04 / +90 4445065 Faks No:+90 432 4865413
e-Posta:yuhadyek@yyu.edu.tr İnternet Adresi:http://www.yyu.edu.tr
Bilgi için: Mehmet Şah OĞUZ
Unvanı: Şef
Tel No: 29019

Bu belge, güvenli elektronik imza ile imzalanmıştır.
