# Supplementary material for: Seroprevalence and risk factors associated with bluetongue and Schmallenberg virus infections in domestic small ruminants in Türkiye
Source: Trop Anim Health Prod. 2026 Mar 16;58(3):181. doi: 10.1007/s11250-026-04951-9 (PMC12992406; doi:10.1007/s11250-026-04951-9)
Supplement: Supplementary file 2 — Supplementary Material 2 [file 11250_2026_4951_MOESM2_ESM.pdf]

T.C.
VAN VALİLİĞİ
İl Tarım ve Orman Müdürlüğü

Sayı : E-44762815-325.04.02-17646968 17.01.2025

Konu : Proje Bazlı İzin (Dr. Öğretim Üyesi Ali Rıza BABAOĞLU)

DAĞITIM YERLERİNE

İlgi : Sağlık Bilimleri Enstitüsü Müdürlüğünün 10.01.2025 tarihli ve 49072339-100.001.001-E. 638200 sayılı yazısı.

Üniversiteniz Veteriner Fakültesi Viroloji Anabilim Dalı Başkanı Dr. Öğretim Üyesi Ali Rıza BABAOĞLU nun yürütücülüğünü yaptığı ve Sağlık Bilimleri Enstitüsü Yüksek Lisans Öğrencisi Feride Firdevs ERTARĞIN' ait tez çalışması olan ***''Van İlindeki Küçük Ruminantlarda Abortus neden olan Bazı Viral Etkenlerin Seroprevalansının araştırılması''*** başlıklı projesi **13.12.2011 tarih ve 28141 sayılı Resmi Gazetede yayımlanarak yürülüğe giren ''Deneysel ve Diğer Bilimsel Amaçlar için Kullanılan Hayvanların Refah ve Korunmasına Dair Yönetmelik''** çerçevesinde incelenmiş olup; Kurumumuzca uygun görülmüştür. Bahse konu çalışmanın yapılmasından en az bir gün önce Müdürlüğümüze bilgi verilmesi, çalışılan alanda Müdürlüğümüz ilgili konu sorumlusu personellerince ve proje sonucunun İl Müdürlüğümüze gönderilmesi hususunda;

Gereğini arz ederim.

İbrahim GÖRENTAŞ
İl Müdürü V.

Dağıtım:

Gereği:
Van Yüzüncü Yıl Üniversitesi Rektörlüğüne (Sağlık Bilimler Enstitüsü)

Bilgi:
Sayın Feride Firdevs ERTARĞIN

Bu belge, güvenli elektronik imza ile imzalanmıştır.
Doğrulama Kodu: 0818DE1C-0B9C-4501-87C9-45570CE17D3D Doğrulama Adresi: https://www.turkiye.gov.tr/tarim-ebys
İskele Cad. Abdurrahmangazi Mah. Çalı Durağı 65040 Tuşba/VAN
Tel: (0432) 222 02 37 Faks: (432)222 0067
E-Posta: van@tarim.gov.tr Kep: van@gthb.hs01.kep.tr
KEP Adresi : tarimveormanbakanligi@hs01.kep.tr
Bilgi için:İsmail Hakkı BEHCET
Veteriner Hekim
32174074 - 32174074 - 32174074
